# Supplementary material for: Identification of photoperiod-induced specific miRNAs in the adrenal glands of Sunite sheep (Ovis aries)
Source: Front Vet Sci. 2022 Jul 22;9:888207. doi: 10.3389/fvets.2022.888207 (PMC9354845; doi:10.3389/fvets.2022.888207)
Supplement: Supplementary file 1 [file Data_Sheet_1.ZIP › Supplementary file/miRDeep2 file.zip!/miRDeep2 file/Novel_Structure/SPLP42A3/Novel_513.pdf]

Provisional ID	:	NC_019483.2_37215
Score total	:	20.8
Score for star read(s)	:	3.9
Score for read counts	:	13.4
Score for mfe	:	1.9
Score for randfold	:	1.6
Score for cons. seed	:	
Total read count	:	38
Mature read count	:	36
Loop read count	:	0
Star read count	:	2

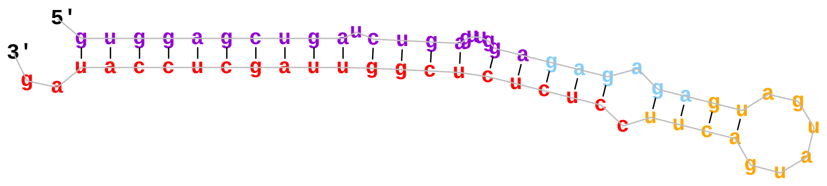

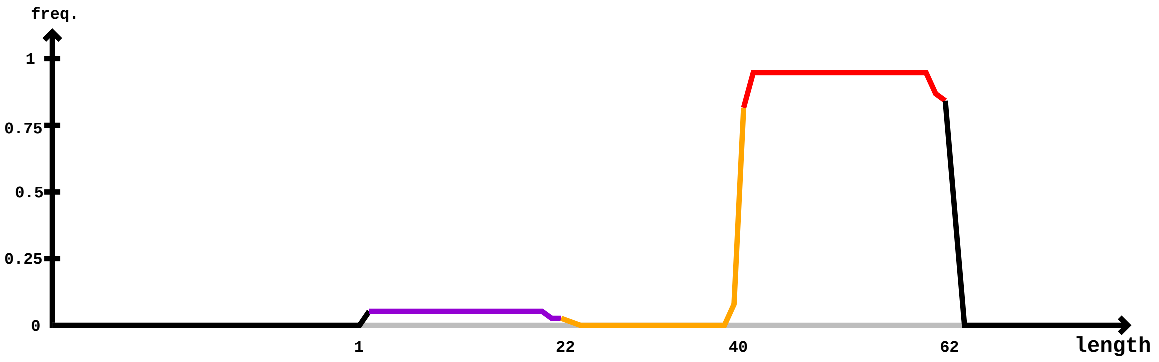


```
                                  Star                                       Mature
5'- uuuacucaguucacugaccgauugugccaguguggagcugaucugaguuggagagagaguaguaugacuuccucucucgguuagcuccauagauggcagaucagccuucu  -3'  obs
    uuuacucaguucacugaccgauugugccaguguggagcugaucugaguuggagagagaguaguaugacuuccucucucgguuagcuccauagauggcagaucagccuucu       exp
    .....((((....))))..((((.(((((.(((((((((((.((((....(((((.((((......)))).))))))))))))))))))))..)))))))))........   reads    mm    sample
    ...............................guggagcugaucugaguug............................................................   1        0     seq
    ...............................guggagcugaucugaguugga..........................................................   1        0     seq
    .....................................................................uccucucucgguuagcuccaua...................   1        0     seq
    .....................................................................uccucucucgguuagcuccauag..................   2        0     seq
    ......................................................................ccucucucgguuagcuccau....................   3        0     seq
    ......................................................................ccucucucgguuagcuccauag..................   22       0     seq
    ......................................................................ccucucucgguuagcuccauaga.................   3        0     seq
    .......................................................................cucucucgguuagcuccauag..................   2        0     seq
    .......................................................................cucucucgguuagcuccauaga.................   3        0     seq
```
